# Interaction of living cable bacteria with carbon electrodes in bioelectrochemical systems

**DOI:** 10.1128/aem.00795-24

**Published:** 2024-07-31

**Authors:** Robin Bonné, Ian P. G. Marshall, Jesper J. Bjerg, Ugo Marzocchi, Jean Manca, Lars Peter Nielsen, Kartik Aiyer

**Affiliations:** 1 Department of Biology, Center for Electromicrobiology, Aarhus University, Aarhus, Denmark; 2 Department of Biology, Center for Water Technology (WATEC), Aarhus University, Aarhus, Denmark; 3 X-LAB, Hasselt University, Agoralaan, Diepenbeek, Belgium; Norwegian University of Life Sciences, Ås, Norway

**Keywords:** cable bacteria, bioelectrochemical system, electrode, extracellular electron transfer, microscopy

## Abstract

Cable bacteria are filamentous bacteria that couple the oxidation of sulfide in sediments to the reduction of oxygen via long-distance electron transport over centimeter distances through periplasmic wires. However, the capability of cable bacteria to perform extracellular electron transfer to acceptors, such as electrodes, has remained elusive. In this study, we demonstrate that living cable bacteria actively move toward electrodes in different bioelectrochemical systems. Carbon felt and carbon fiber electrodes poised at +200 mV attracted live cable bacteria from the sediment. When the applied potential was switched off, cable bacteria retracted from the electrode. qPCR and scanning electron microscopy corroborated this finding and revealed cable bacteria in higher abundance present on the electrode surface compared with unpoised controls. These experiments raise new possibilities to study metabolism of cable bacteria and cultivate them in bioelectrochemical devices for bioelectronic applications, such as biosensing and bioremediation.

**IMPORTANCE:**

Extracellular electron transfer is a metabolic function associated with electroactive bacteria wherein electrons are exchanged with external electron acceptors or donors. This feature has enabled the development of several applications, such as biosensing, carbon capture, and energy recovery. Cable bacteria are a unique class of long, filamentous microbes that perform long-distance electron transport in freshwater and marine sediments. In this study, we demonstrate the attraction of cable bacteria toward carbon electrodes and demonstrate their potential electroactivity. This finding enables electronic control and monitoring of the metabolism of cable bacteria and may, in turn, aid in the development of bioelectronic applications.

## INTRODUCTION

The ability of certain microorganisms to perform extracellular electron transfer (EET) by reducing a solid, external electron acceptor is a unique strategy for energy generation when faced with a lack of soluble electron acceptors in the immediate environment. These microbes, termed variously as electricigens, exoelectrogens, anode-respiring, or electroactive bacteria, use specialized mechanisms either involving redox-active outer membrane cytochromes (1), conductive nanowires (2), or soluble redox shuttles to mediate EET (3). Bioelectrochemical systems (BESs) exploit the respiratory ability of electroactive bacteria to convert chemical energy to electricity (or vice versa). Microbial oxidation of electron donor compounds provided as fuel generates electrons, which are subsequently captured by the electrode and flow in a circuit, resulting in electric current. BESs have provided a wealth of information about different modes of EET, especially in model electroactive bacteria, such as *Geobacter* (4, 5) and *Shewanella* (3, 6). In recent years, they have also provided insights into the fundamental nature of electroactivity in different classes of microbes, including Gram-positive bacteria (7, 8), pathogenic bacteria (9–11), and weak electricigens (12–14). On the applied side, BESs have branched out into several applications, including wastewater treatment (15, 16), heavy metal recovery (17, 18), biosensing of hazardous chemicals (19, 20), powering remote sensors (21, 22), and even monitoring and removal of viral antigens in a medical context (23).

The discovery of cable bacteria has added a new layer of complexity to microbial electron transport processes (24, 25). Cable bacteria are present in the environments where electron donors and acceptors are spatially separated by centimeter distances (26, 27). To complete metabolic reactions for energy generation, cable bacteria utilize long-distance electron transport (LDET) to couple two redox half-reactions over distances far exceeding cell length (27, 28). Found ubiquitously in freshwater and marine sediments (29, 30), cable bacteria oxidize sulfide in the anoxic zone and transport the electrons via conductive periplasmic proteins to the upper sediment layers to reduce oxygen or nitrate (25, 31). A single cable bacterium filament may consist of thousands of individual cells, which may be up to 5 cm in length (26). A unique phenomenon associated with cable bacteria is the presence of other “flocking” bacteria around them, presumably for reducing cable bacteria via interspecies electron transfer (32). This warrants further investigation into whether there are mechanisms developed by cable bacteria for performing EET under certain conditions. Cable bacteria would be able to potentially benefit from EET in environments where oxygen and nitrate are unavailable. EET could also serve as a mechanism for cable bacteria to interact electrically with other microorganisms.

Recently, cable bacteria were found on the anodes of a benthic microbial fuel cell, raising speculations regarding their ability to interact with electrodes in anoxic conditions (33, 34). However, further research probing this aspect has been lacking. Despite the fact that periplasmic wires extracted from cable bacteria maintain their conductivity when connected between two electrodes (35), systematic evidence for living cable bacteria to interact with electrodes or perform EET is still missing. A challenge currently faced in this aspect is that cable bacteria are not yet isolated in pure culture. In this study, we visualize and quantify the freshwater cable bacterium *Electronema aureum GS* around carbon electrodes in BESs. *E. aureum* GS is the best-studied strain of cable bacteria available, with stable single-strain cultures enabling the discovery of phenomena like “flocking” (32, 36, 37). We demonstrate the interactions of *E. aureum* GS with carbon electrodes in two different BES setups. The first conventional three-electrode cell allowed for amperometry, voltammetry, qPCR, 16S rRNA gene sequencing, and scanning electron microscopy (SEM) to determine the presence and impact of cable bacteria on electrodes. Another BES was specifically designed for cable bacteria to study their interaction with electrodes in real time using non-invasive microscopy. Our data suggests that the electrode is a viable alternative electron acceptor for cable bacteria, although the mechanism remains to be elucidated. This finding could help tap into the potential of cable bacteria to develop various bio-electric applications, while also presenting an opportunity to explore fundamental aspects of the electron transfer process and metabolism.

## RESULTS AND DISCUSSION

### Current generation by the cable bacteria-enriched cultures

**Fig 1:**
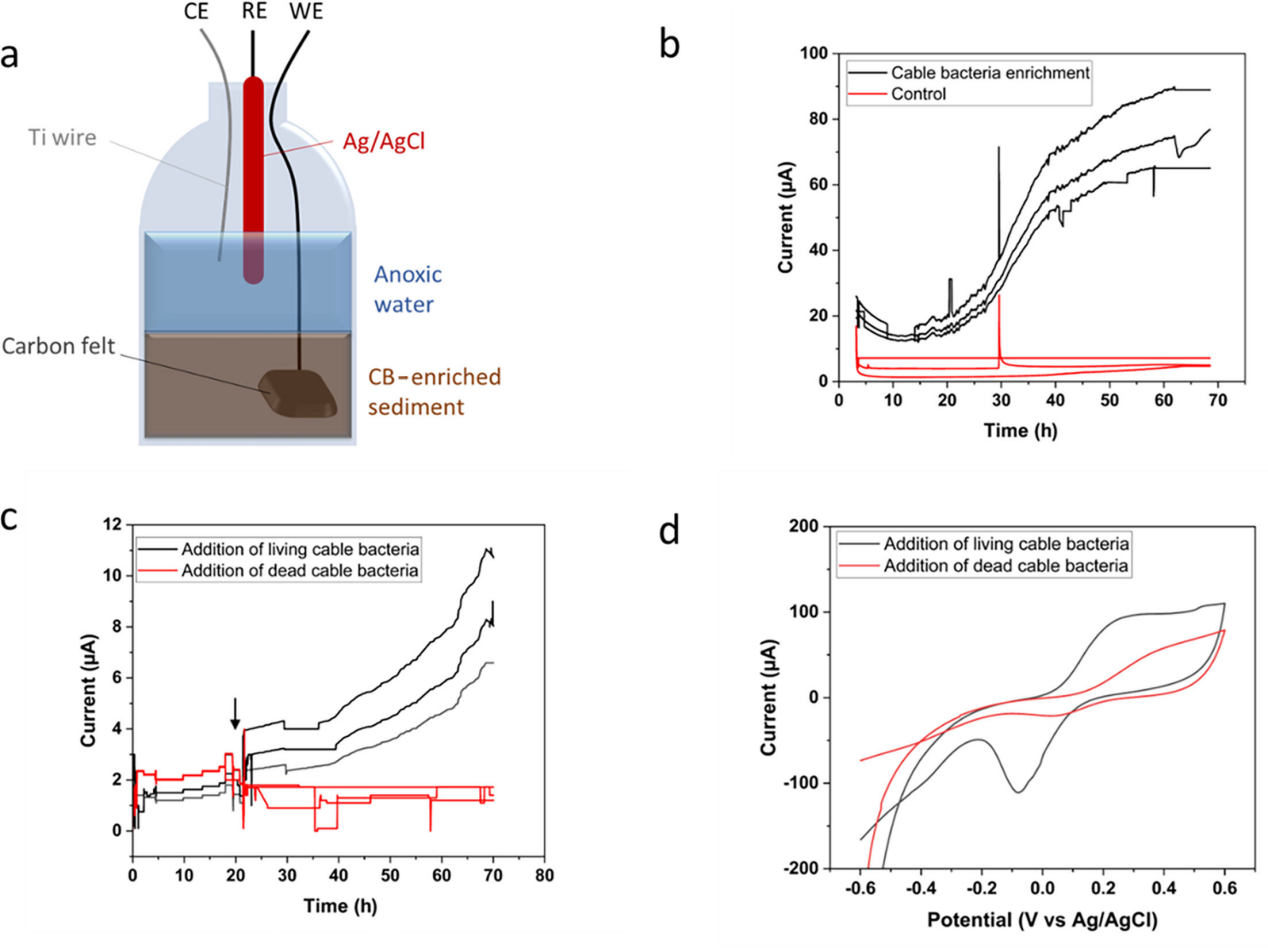
(a) Schematic representation of the sediment BES inoculated with CB-enriched sediment. (b) Chronoamperometry results show a rising current for the CB-enriched sediment within 24 h (black), whereas the autoclaved sediment control shows stable currents below 5 µA (red). The data from triplicate reactors are presented. (c) When isolated cable bacteria are added to autoclaved sediment (indicated by the arrow), the current rises significantly within a day. (d) Cyclic voltammograms (average from three reactors) performed after experiment 1c show a redox-active behavior. A scan rate of 1 mV/s was used.

To investigate the potential electroactivity of cable bacteria, a three-electrode cell consisting of a carbon felt working electrode, Ag/AgCl reference electrode, and a Ti counter electrode was inoculated with freshwater sediment enriched with the single-strain *E. aureum* GS (Fig. 1a). No other cable bacterium strains were present. After inoculation, the current demonstrated a sigmoidal increase (Fig. 1b), with average values ranging between 17 and ~78 µA. This indicates the capability of the cable bacteria consortium to reduce the electrode via EET. The control cells with autoclaved sediment produced a steady background current of 4.75 ± 0.5 µA throughout the incubation. Furthermore, the pH of the overlying water in the inoculated cells decreased over time, from 7.4 to 6.7 (Table S1), whereas the pH of the control cells did not change. These changes arise as a result of the mass transfer of protons after biological substrate oxidation, which may become less effective over long-term operation (38, 39).

To further elucidate the specific contribution of cable bacteria to the overall measured current, another set of three-electrode cells was inoculated with autoclaved sediment, to which approximately 10 clean cable bacteria filaments were added after fishing them from the sediment using sterilized glass hooks. Within 48 h after the addition of living cable bacteria, the current increased from ~2 to ~8 µA (Fig. 1c), suggesting that very few cable bacteria are required to see the electrochemical effect. The experiment was stopped after 72 h for further analysis. The addition of heat-killed cable bacteria to autoclaved sediments did not increase the current. The results suggest that cable bacteria reduce the electrode in the absence of conventional electron acceptors, such as oxygen.

Figure 1d demonstrates the cyclic voltammograms for the above experiment when cable bacteria are added to the autoclaved sediment. Prominent redox peaks are observed at 0.22V and −0.05V. The results indicate that it is a biotic-mediated reaction as heat-treated cable bacteria did not generate the redox peaks. Although the contribution of other electroactive bacteria present in the inoculum cannot be excluded, the peaks do not seem to be associated with outer membrane cytochromes typically seen for *Geobacter* (40) and could shed light on a potential mechanism for EET. *Geobacter* CVs demonstrate peaks at negative potentials, demonstrating catalytic activity of cytochrome redox centers in electron transfer (40, 41). Scanning the enrichments for potential mediators could prove useful toward determining mediated electron transfer mechanisms.

### qPCR, 16S rRNA gene sequencing, and SEM reveal an increased presence of cable bacteria on the poised electrode

The above experiments suggest that cable bacteria contribute to the current in the three-electrode cell. However, it is essential to note that other electroactive bacteria could also have been transferred along with cable bacteria in this process. Therefore, further experiments were set up to study specific interactions between cable bacteria and electrodes.

**Fig 2:**
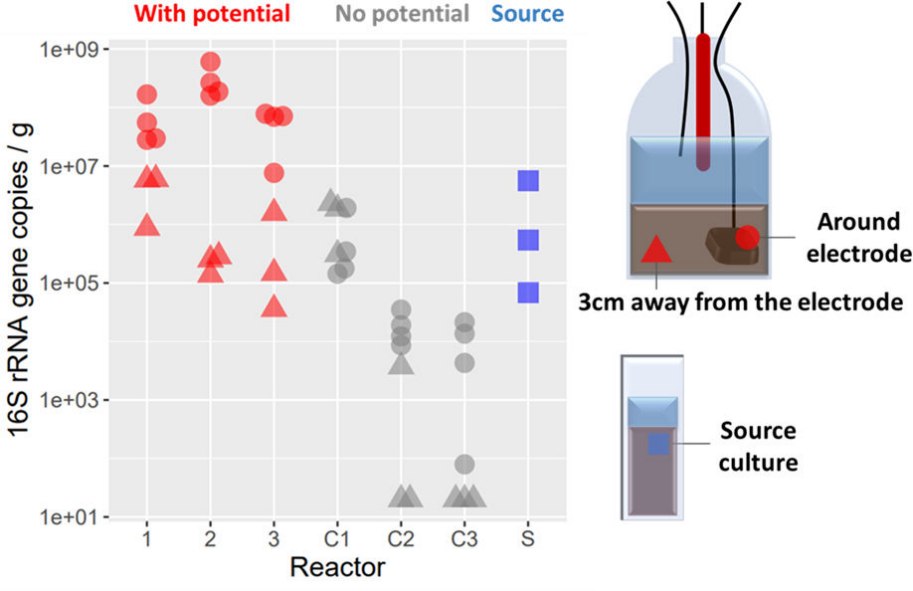
qPCR performed on the electrode reveals an increased abundance of *Electronema aureum* GS on the electrode surface per gram of sediment. Reactors C1, C2, and C3 are the controls (without applied potential), whereas 1, 2, and 3 are the experimental reactors. The source culture indicates the freshwater sediment containing the single-strain enrichment of *E. aureum* GS.

To assess whether the cable bacteria were abundant on the electrodes, qPCR and 16S rRNA gene sequencing were performed on the carbon felt working electrodes. qPCR was performed at the completion of the experiment after 72 h, both on the carbon felt working electrodes and sediment approximately 3 cm away from the electrode. Both samples were taken in triplicates. The number of cable bacterial gene copies was significantly higher on the electrode surface compared with the sediment (Fig. 2). Cable bacteria were on average 490 times more abundant at the poised electrode compared with the original sediment, and about 640 times more abundant compared with the unpoised carbon felt electrode in the controls (C1, C2, and C3). With the applied potential, they were 70 times more abundant at the poised electrode than in the bulk sediment.

In the open-circuit negative controls C2 and C3, a similar trend was observed. Control C1 however showed higher abundance and deviated from the trend, which could be due to accidental oxygen leakage into the reactor.

The 16S rRNA gene fractional abundance of the top ten genera enriched in the three-electrode cell is presented in Fig. S1. 16S rRNA gene sequencing corroborated the qPCR data and interestingly revealed *E. aureum* GS to be the second-most abundant species on the poised electrodes after *Geobacter* (Fig. S1). No *E. aureum* GS was detected on the electrode of the control reactors without an applied potential.

To demonstrate that the cable bacteria attached to the electrodes were living filaments, a specialized setup known as the trench slide was used. A trench slide is a microscopy slide with a central cavity that is filled with sediment containing cable bacteria (27). Living cable bacteria stretch out from the sediment toward the edge to access oxygen and can be visualized via microscopy. The trench slides were filled with the sediment used in the three-electrode cell to screen the activity of cable bacteria adhered onto the carbon felt electrodes as described above. One set of trench slides (*n* = 3) was inoculated with sediment collected on the electrode surface and in the immediate vicinity of the electrodes, whereas a second set (*n* = 3) was inoculated with sediment collected approximately 3 cm away from the electrode. A third set of trench slides was inoculated with sediment from an identical control setup where no potential was applied. Living cable bacteria filaments were observed only in the sediment collected near the electrode (Fig. S2) when a potential was applied, further substantiating the qPCR results.

**Fig 3:**
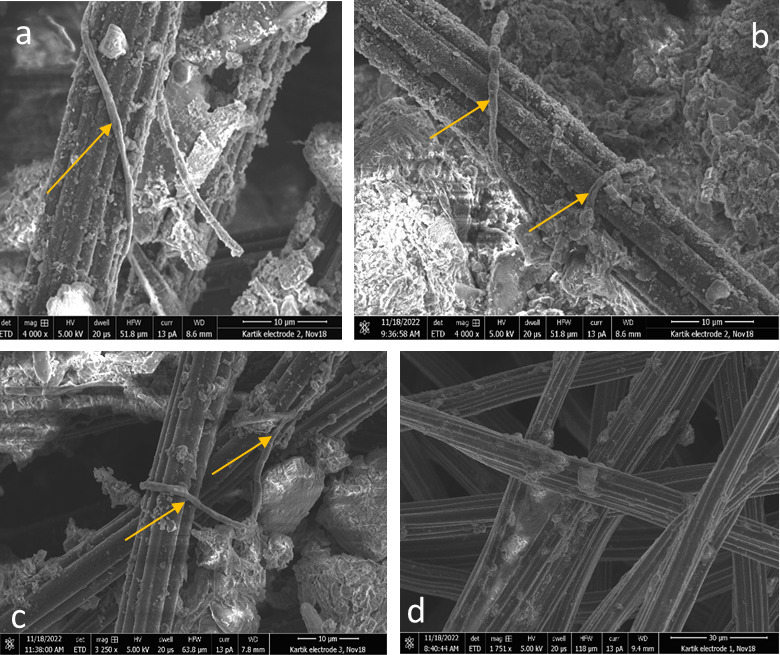
Scanning electron microscopy of the electrodes from the CB-inoculated sediment BES . Images a–c show the presence of cable bacteria on the electrodes (indicated with yellow arrows), whereas d is the negative control (electrode without applied potential).

To understand whether cable bacteria directly interacted with the electrode, we investigated the carbon felt working electrodes from these experiments with scanning electron microscopy (SEM) (Fig. 3). Cable bacteria were visible on all of the tested samples originating from the poised anodes (Fig. 3a through c). Some cable bacteria were coiled around the carbon felt electrode, possibly to maximize contact area with the electrode. The cable bacteria present on the electrodes had lengths exceeding 150 µm. The individual cells within the cable bacteria were ~2 µm in length, and cell–cell junctions were clearly observed in the SEM images, along with potential mineral deposits. For the negative control, where the electrodes did not have an applied potential, cable bacteria were not found on the electrode surface (Fig. 3d).

### Real-time attachment of cable bacteria to electrodes using light microscopy

**Fig 4:**
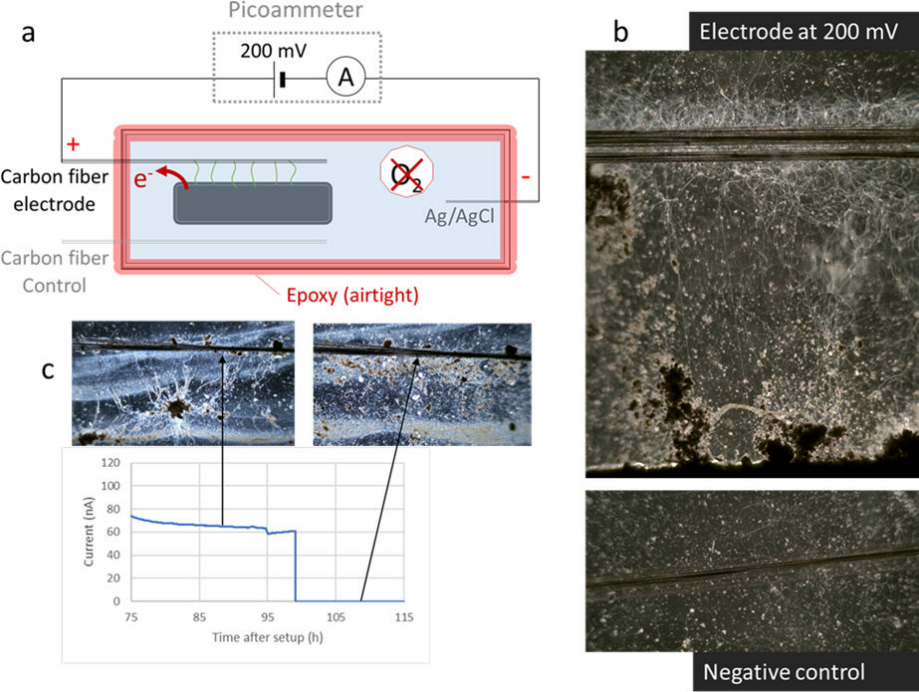
(a) A two-electrode electrochemical cell on a microscopy slide (with a chlorinated silver wire as a pseudoreference and counter electrode) makes it possible to study real-time interactions with a carbon fiber electrode. An unpoised carbon fiber electrode served as the control. (b) Cable bacteria populate the poised carbon electrode as a white filamentous mass, with some of them crossing between the sediment and the electrode. The unpoised carbon fiber control did not contain cable bacteria. (c) When the potential is switched off at the carbon electrode, cable bacteria retracted from the electrode surface.

To observe the response of cable bacteria to a switchable applied potential in real time, a different configuration of the microbial electrochemical cell was constructed within a trench slide. Carbon fiber (CF) electrodes were glued parallelly on either side of the trench to function as the working and counter electrodes, and an Ag/AgCl pseudo-reference electrode was introduced 3 cm away from the trench (Fig. 4a). The edges of the trench slide were sealed to block oxygen diffusion in the trench slide, leaving the CF electrode as the sole electron acceptor. The bacteria started populating the poised CF electrode within a few hours after inoculation (supplementary, Video S1) along with simultaneous generation of the current. By contrast, no cable bacteria were observed at an unpoised CF electrode (Fig. 4b). Although most of the cable bacteria aggregated as white filamentous mass around the electrode, some retained connection to the sediment in the middle (see Video S2 and S3 to compare the poised and unpoised carbon fibers). One interpretation of this result is that the cable bacteria with one end in the sediment had privileged access to sulfide in the sediment, and another is that they just accidentally had an end trapped in the sediment as sufficient sulfide diffused out of the sediment, giving the aggregated cable bacteria easy access to an electron donor.

In a different trench slide reactor of the same configuration, the current was monitored over 100 h of operation and showed a gradually increasing current that reached values between 50 and 100 nA (data not shown). The poised electrode contained several cable bacteria at the electrode stretching from the sediment (Fig. 4c). After 98 h of operation, the applied potential was switched off, and cable bacteria retracted from the electrode in 10 h (Fig. 4c). This indicates that cable bacteria stay alive and active in an anoxic environment for days in the presence of a solid electron acceptor. Similarly, when the unpoised electrode was poised after 5 days of incubation, cable bacteria reappeared on the electrode after approximately 2 h (Fig. S3), indicating their ability to survive the absence of soluble electron acceptors. The behavior of cable bacteria in moving toward an electrode in the presence of an applied potential could serve as a mechanism to effectively use alternative electron acceptors, aiding survival in oxygen-deficient environments (42, 43).

The open question remains whether cable bacteria make direct contact with the electrode or reduce the electrode through mediators. The first indications of a direct connection can be seen through a timelapse of cable bacteria interacting with electrodes, where they seem to pull on the carbon fibers over time (Video S4). Additionally, when the microscopy slide was subjected to agitation during operation, the bacteria retained their connection with the carbon fibers, indicating the robustness of their connection to the electrode (Video S5).

### Potential mechanism of EET

The attraction of cable bacteria toward electrodes only in the presence of an applied potential and the consequent increase in current production suggests that cable bacteria actively interact with the electrode. The mechanism of potential EET to the electrode remains an open question, despite supporting evidence for direct and mediated electron transfer. On the one hand, the cyclic voltammetry results indicate the presence of redox mediators. On the other hand, SEM and microscopy results of cable bacteria coiled around the electrode, together with previous results from Reimers et al. (34), allude to direct electron transfer. Several cytochromes recently identified in cable bacteria (27, 44) could also potentially be implicated in extracellular electron transfer, although their structural and functional role remains to be elucidated. Cable bacteria have not yet been isolated in pure culture and the single-strain enrichments in sediments constitute a “mixed” community. *Geobacter* was also enriched along with *E. aureum* GS on the electrodes. Although some of the current generated is certainly due to *Geobacter*, the presence of cable bacteria on electrodes and the qPCR results hint at the possibility of EET by cable bacteria.

In the natural environment, the main physiological relevance of EET could be to help cable bacteria to survive conditions where oxygen and nitrate are either depleted or unavailable. Iron and manganese-based minerals are amply present in the sediment, which could potentially be used as terminal electron acceptors under oxygen/nitrate-limiting conditions. *Vibrio natriegens*, recognized to be among the fastest respiring microbes due to their rapid oxygen consumption, has also evolved a strategy to perform EET under low oxygen conditions (45). Such a hybrid model could similarly benefit cable bacteria and aid in their survival under anaerobic conditions. The response of cable bacteria to soluble redox shuttles mediating electron transfer also needs to be investigated.

Overall, these findings indicate that cable bacteria tend to migrate toward an electrode under an applied potential and potentially reduce it, thereby generating electric current. However, it is not yet clear whether cable bacteria can actively divide and grow in anoxic conditions over long periods of time.

### Conclusions

Cable bacteria were found to be attracted to carbon electrodes and remain active in response to an applied potential in different bioelectrochemical systems. This work paves the road for similar bioelectrochemical experiments to elucidate the magnitude and mechanism of electron transfer from cable bacteria to the electrode as well as the behavioral triggers and any associated growth. The successful growth of cable bacteria in bioelectrochemical systems will enable the development of applications, such as bioremediation of soil contaminants such as petroleum hydrocarbons (46, 47) and biosensing. The addition of cable bacteria to an electroactive consortium could potentially be used to produce chemicals from electricity.

## MATERIALS AND METHODS

### Construction of the three-electrode cell

Three-electrode cells were constructed from glass serum bottles (70 mL volume). The inoculum consisted of single-strain enrichment of *E. aureum* GS (36) in freshwater sediment as described by Thorup et al. (37), with other associated bacteria present in the sediment. Subsequently, 10 g of sediment was weighed and added into the glass bottle, followed by the addition of N_2_-sparged anoxic lake water. For the controls, the sediment was autoclaved for 40 min at 121°C before use in the three-electrode cell. Sulfide, organics, and dissolved inorganic carbon are all naturally available in the sediment as electron donors and carbon sources, and no compound was externally added. The working electrode consisted of carbon felt (Thermo Fisher, dimensions 2 cm × 2 cm × 0.3 cm), a commercial Ag/AgCl electrode (REF201 Radiometer Analytical, Denmark) constituted the reference electrode, and a titanium wire (Sigma) was used as the counter electrode. The working electrode was buried 2 cm in the sediment, whereas the reference and counter electrodes were positioned in the overlying water (Fig. 1a) separated by approximately 1 cm. The electrodes were connected to a potentiostat (MultiSens 4, PalmSens, Netherlands), and a potential of 200 mV was applied to the working electrode (Fig. 1b). In an additional set of experiments (Fig. 1c), a similar three-electrode setup was prepared with autoclaved sediment. Around 10–20 cable bacteria were extracted with glass hooks from natural freshwater sediment, cleaned in milli-Q water, and added to the cell. Cyclic voltammetry was performed before and after cable bacteria addition. The potential window was −0.6V to +0.6V at a scan rate of 10 mV s^−1^. Data were recorded using the MultiTrace software.

### qPCR

For qPCR, primers specific to the *E. aureum* GS strain were used (GS68F: 5′-GGTAGTTTCCTTCGGGGGAC-3′, GS178R: 5′-CTTCGGCAATGCGGCGTATC-3′), which were designed based on the 16S rRNA gene for strain from its genome using the Arb PROBE DESIGN tool (48) with the Silva Ref NR 99 database version 132 (49). Note that these primers are designed specifically for the GS strain and will likely not amplify DNA from other *Electronema* strains and species. DNA was extracted from sediment and electrode material (0.5 g per sample) using the DNeasy PowerLyzer PowerSoil Kit (Qiagen) following the manufacturers’ instructions. Quantification of cable bacteria was performed using quantitative PCR with 2 µL of DNA extract added to each 20-µL reaction along with RealQ Plus 2× Master Mix Green (Ampliqon, Odense, Denmark), 0.1% BSA, 0.5 pmol/µL of each primer. A synthetic standard containing base positions 1–200 from the 16S rRNA gene sequence was used for the standard curve (GenScript, Rijswijk, Netherlands). The qPCR was performed on a Strategene Mx3005P qPCR machine (Agilent Technologies, Santa Clara, CA, USA) with an initial 15-min activation step at 95°C, followed by 45 cycles with 30 s at 95°C, 30-s annealing at 60°C, 20-s elongation at 72°C, and 15-s acquisition at 95°C. A melting curve from 60°C to 95°C confirmed the absence of secondary amplicons. Abundance was calculated per gram of sediment.

### 16S rRNA gene sequencing

At the conclusion of the experiments, 16S rRNA gene sequencing was done to determine the composition of the microbial community from the three-electrode cells. Samples constituted the electrode surface, with sediment approximately 3 cm from the electrode and the original inoculum source containing *E. aureum* GS. V3/V4 fragments of 16S rRNA genes were amplified from DNA extracts in a PCR using primers Bac341F (GGGCATCATGATGCGCCTG) and Bac805R (TCRACCTTCTCGCACTTCCA) (50) at 0.2 pmol/µL using 2X KAPA HiFi Hotstart ReadyMix (Roche, Switzerland) in a 25-µL reaction containing 2.5 µL of template DNA. PCR was performed with an initial 15-min activation step at 95°C, followed by 30 cycles of 30 s of denaturation at 95°C, 30 s of annealing at 54°C, and 20 s of elongation at 72°C, followed by a final finishing step for 5 min at 72°C. This PCR was repeated with primers containing adapter sequences (Bac341F + adapt TCGTCGGCAGCGTCAGATGTGTATAAGAGACAGCCTACGGGNGGCWGCAG, Bac805R + adapt GTCTCGTGGGCTCGGAGATGTGTATAAGAGACAGGACTACHVGGGTATCTAATCC), and only 15 cycles were used. A final PCR was used to add indices, carried out as the initial PCR, but using an annealing temperature of 55°C. Index primers from the Nextera XT Index Kit (Illumina, San Diego, CA, USA) were used. Prepared sequencing libraries were sequenced on an Illumina MiSeq DNA sequencer using the V3 sequencing kit.

Primer sequences were removed from sequencing reads using cutadapt version 3.5 (51). Reads were clustered into amplicon sequencing variants (ASVs) and classified using dada2 version 1.28.0 (52) in R version 4.3.2, with default filtering and trimming settings used apart from forward reads being trimmed at 250 bases and reverse reads at 200 bases. Classification was performed using the Silva 138.1 prokaryotic SSU database (49, 53).

### Scanning electron microscopy (SEM)

After the conclusion of the experiment, a 1-cm² piece of the carbon felt working electrode was cut off carefully and placed on a stopper for SEM analysis. The instrument consisted of a dual-beam FIB/SEM 2D Versa (Thermo Fisher). Acquisition parameters included a high vacuum of 5 kV, a working distance of 10 mm, and a current of 13 pA.

### Construction of the trench slide three-electrode cell

In addition to the above setup, a bioelectrochemical cell was constructed within a trench slide system (Fig. 4a). The trench slide was a glass slide containing a central, 1-mm deep, rectangular cavity for inoculating sediment (27). Two bundles of carbon fiber (CF) wires (10-µm diameter, T300SC, Toray, Japan) were glued parallelly on each side of the trench at a distance of 5 mm. The CF wire on one side functioned as the working electrode, whereas the one on the other side was left unpoised as a negative control. At a distance of 20 mm from the trench, a chlorinated Ag wire was introduced to function as the pseudo-reference and counter-electrode. The central trench was filled with sediment enriched with *E. aureum* GS, followed by addition of N_2_-sparged lake water over the sediment. A coverslip was carefully placed on top, and the trench slide was incubated at 25°C for 4 h to enable cable bacteria to come out from the sediment. After incubation, epoxy (Loctite instant mix, Henkel, USA) was applied around the edges of the coverslip to block oxygen penetration into the trench. Using a picoammeter (Unisense, Denmark), a potential of 200 mV was applied between the working electrode and the Ag wire, and the resulting current was recorded using SensorTrace software (Unisense).

### Microscopy of the carbon fiber electrode

The trench slide BES was investigated via confocal microscopy (Zeiss Observer Z1, Zeiss) to study the attachment of cable bacteria onto the electrodes.

## Data Availability

16S rRNA gene amplicon sequencing data are available from the NCBI Sequence Read Archive under BioProject number PRJNA1044556. All other data are included in the article and its supplemental material.

## SUPPLEMENTAL MATERIAL

Figure S1 aem.00795-24-s0001.tif 16S rRNA gene fractional abundance of the most abundant genera enriched in the three-electrode cell.

Figure S2 aem.00795-24-s0002.tif Migration of cable bacteria toward the electrode.

Figure S3 aem.00795-24-s0003.tif Carbon fiber electrode before and after applying a potential.

Supplemental material aem.00795-24-s0004.docx Table S1, Figures S1 to S3, and Videos S1 to S5.

ASM does not own the copyrights to Supplemental Material that may be linked to, or accessed through, an article. The authors have granted ASM a non-exclusive, world-wide license to publish the Supplemental Material files. Please contact the corresponding author directly for reuse.
