# Supplementary figures and images for: Interaction of living cable bacteria with carbon electrodes in bioelectrochemical systems
Source: Appl Environ Microbiol. 2024 Jul 31;90(8):e00795-24. doi: 10.1128/aem.00795-24 (PMC11337825; doi:10.1128/aem.00795-24)

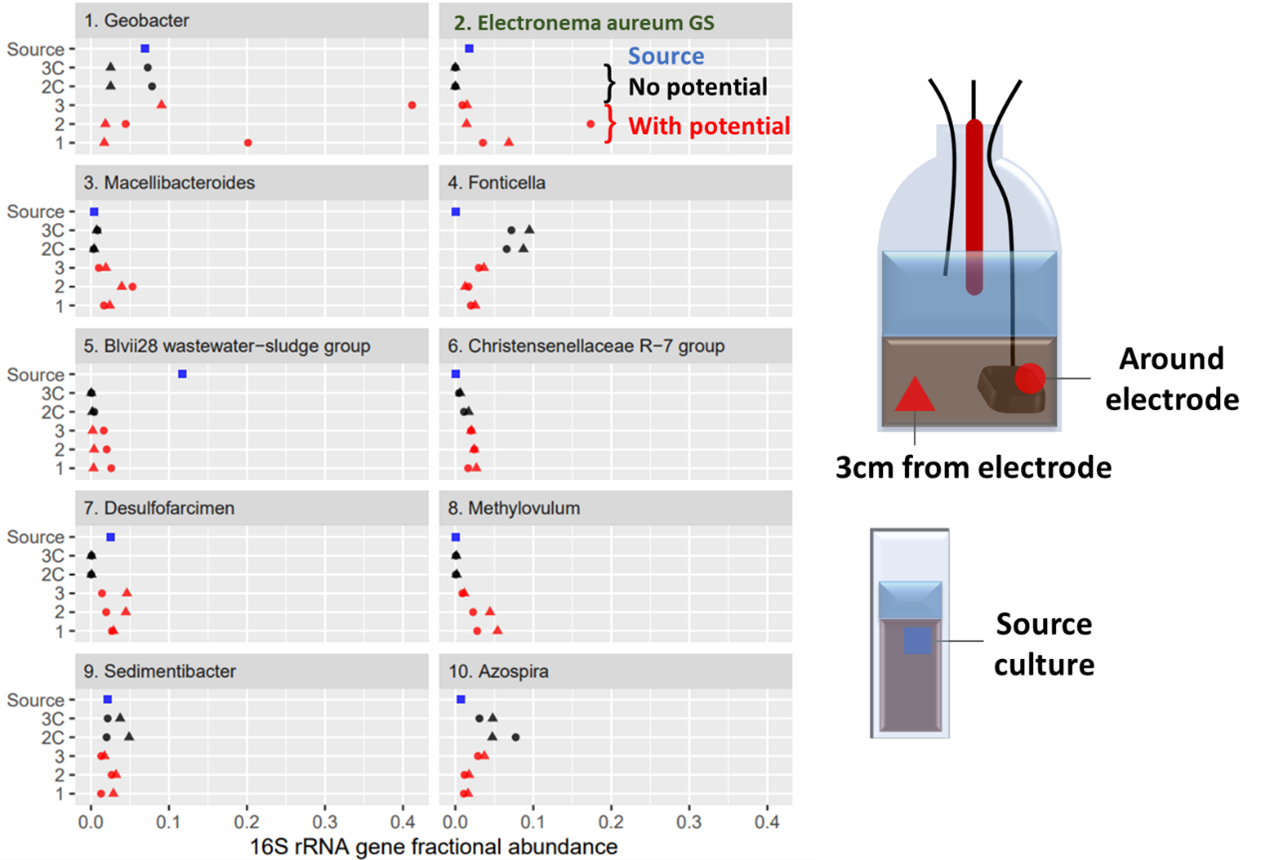

Supplement: Figure S1 — 16S rRNA gene fractional abundance of the most abundant genera enriched in the three-electrode cell. [file aem.00795-24-s0001.tif]

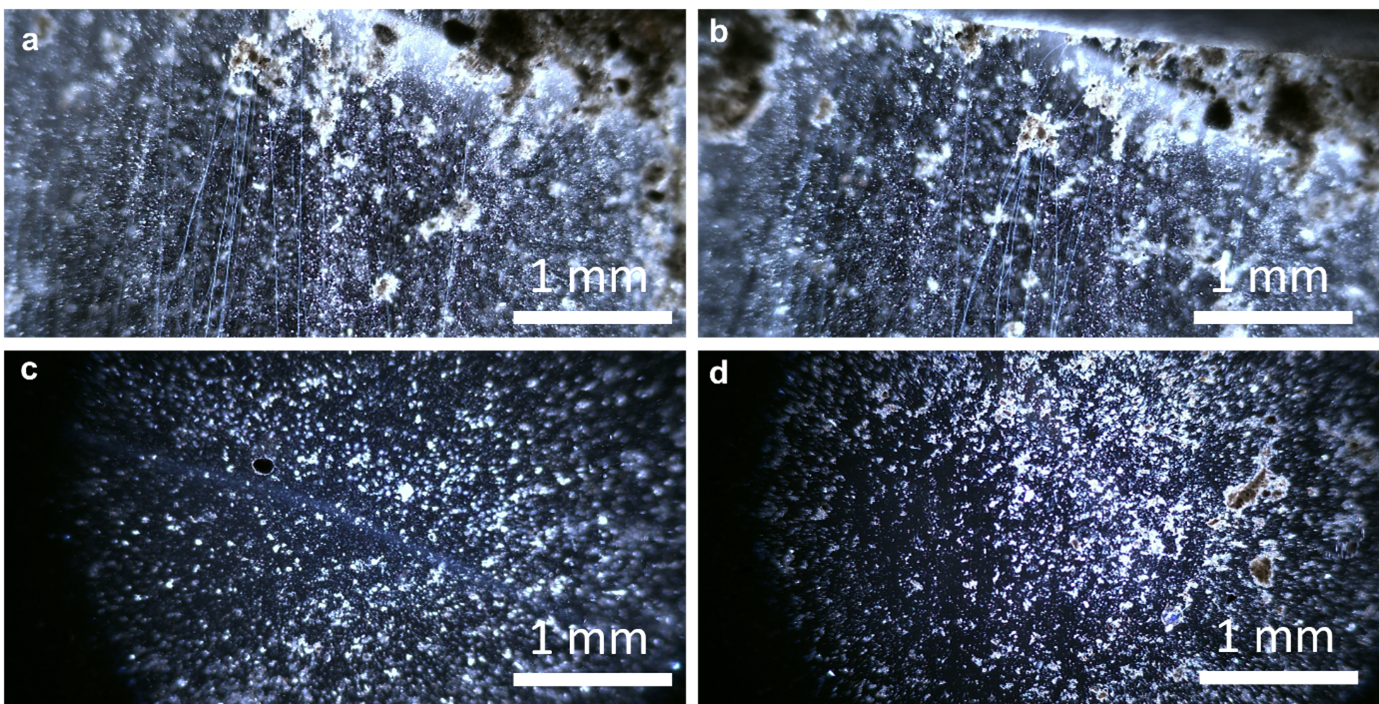

Supplement: Figure S2 — Migration of cable bacteria toward the electrode. [file aem.00795-24-s0002.tif]

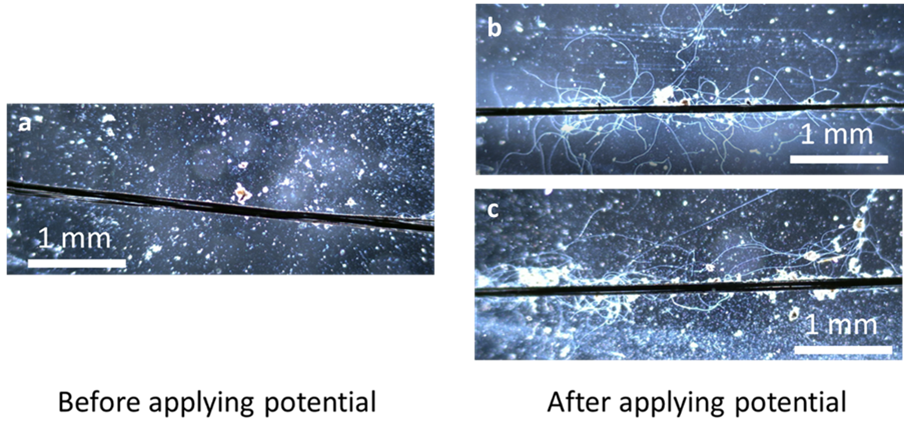

Supplement: Figure S3 — Carbon fiber electrode before and after applying a potential. [file aem.00795-24-s0003.tif]
