# Supplementary material for: Interaction of living cable bacteria with carbon electrodes in bioelectrochemical systems
Source: Appl Environ Microbiol. 2024 Jul 31;90(8):e00795-24. doi: 10.1128/aem.00795-24 (PMC11337825; doi:10.1128/aem.00795-24)
Supplement: Supplemental material — Table S1, Figures S1 to S3, and Videos S1 to S5. [file aem.00795-24-s0004.docx]

**Supplementary Information**

Interaction of living cable bacteria with carbon electrodes in bioelectrochemical systems

Robin Bonné^1^, Ian P.G. Marshall^1^, Jesper J. Bjerg^1^, Ugo Marzocchi^1,2^, Jean Manca^3^, Lars Peter Nielsen^1^, Kartik Aiyer^1 *^

^1^Center for Electromicrobiology, Department of Biology, Aarhus University, Aarhus, Denmark

^2^Center for Water Technology (WATEC), Department of Biology, Aarhus University, Aarhus, Denmark

^3^X-LAB, Hasselt University, Agoralaan D, B-3590 Diepenbeek, Belgium

*Corresponding author: [kartikaiyer@bio.au.dk](mailto:kartikaiyer@bio.au.dk)

| Time (hours) | pH in the overlying water in CB-inoculated BES | pH in the overlying water in control BES |
| --- | --- | --- |
| 0 | 7.4 | 7.4 |
| 24 | 7.1 | 7.3 |
| 48 | 6.9 | 7.5 |
| 72 | 6.7 | 7.4 |

Table S1: pH changes in the overlying water of the three-electrode cell. The pH reduced due to the electrochemical activity of the microbes, leading to mass transfer of protons.


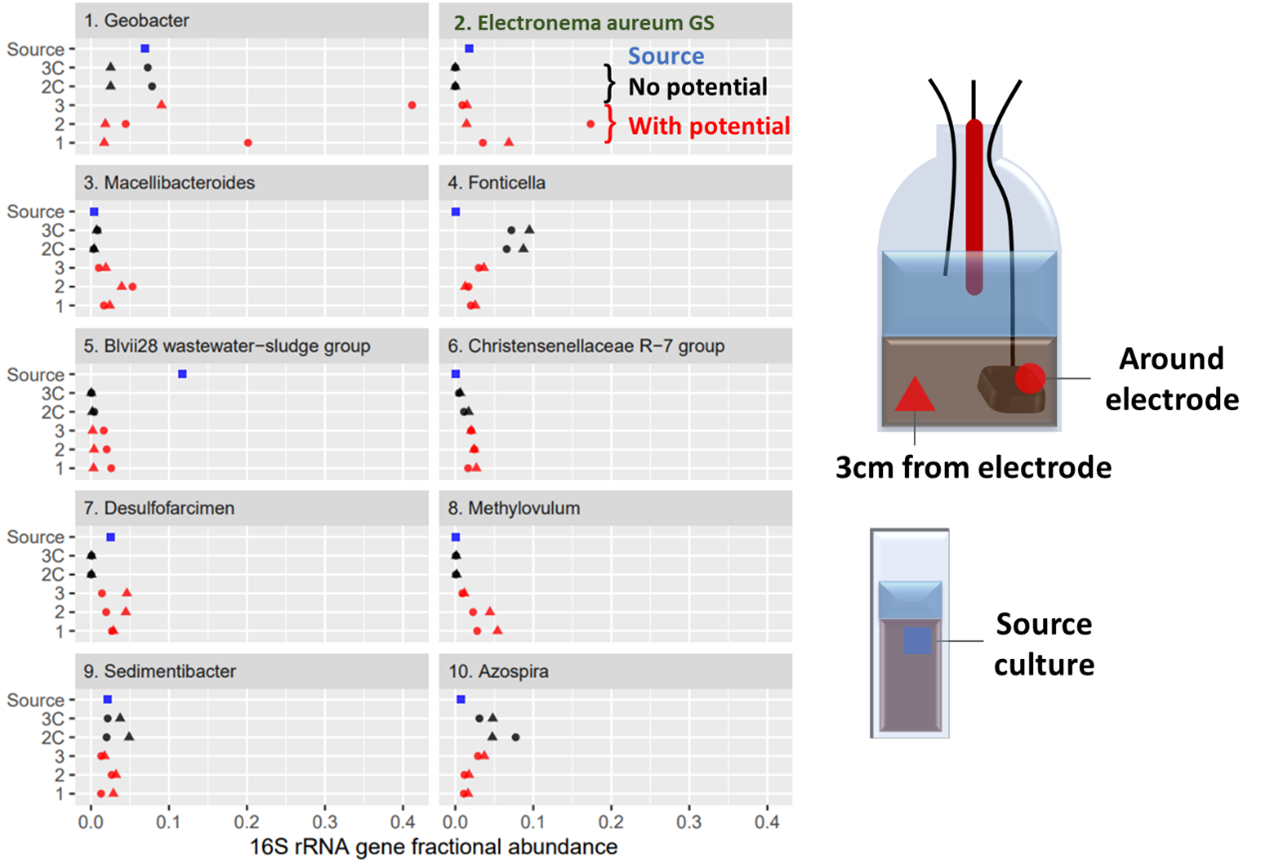


Fig S1: 16S rRNA gene fractional abundance of the most abundant genera enriched in the three-electrode cell. The top ten genera for the community around the poised electrode are depicted here, showing *Electronema aureum* GS to be the second most enriched genus after *Geobacter*. With an applied potential, the abundance of *Electronema aureum* GS was higher on the electrode surface. Reactors without potential did not reveal the presence of *Electronema aureum* GS on the electrode.


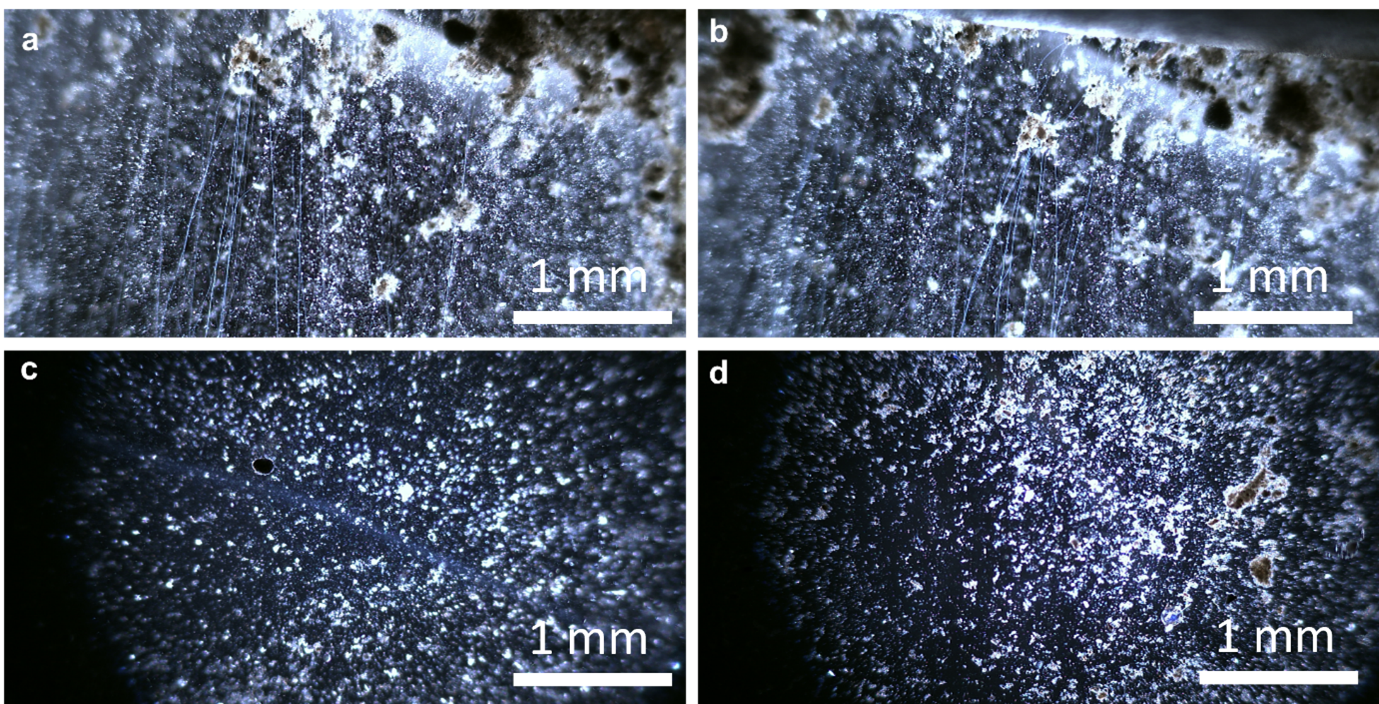


Figure S2: Migration of cable bacteria towards the electrode. After the classical BES experiment, the sediment on the electrode and away from the electrode was used to prepare trench slides. Cable bacteria were present in the sediment at the electrode (a, b), while no cable bacteria were detected in sediment away from the electrode (c, d).


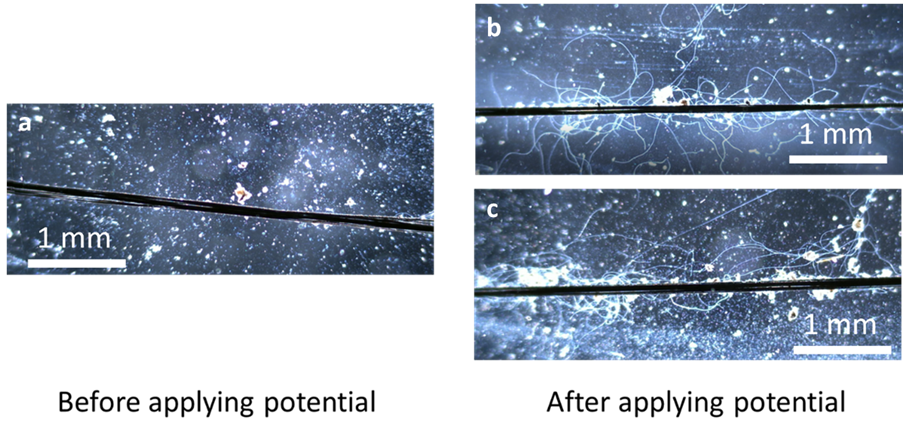


Figure S3: The carbon fibre electrode before and after applying a potential. The poised electrode attracts cable bacteria.

**Supplementary Video links**

Video 1: [Cable bacteria populating the poised electrode](https://www.dropbox.com/s/r8v4kt0alwir7i7/VideoS1_CEBBESpaper_AiyerBonn%C3%A9_PopulatingPoised3.mp4?dl=0)

Cable bacteria populate the electrode: Cable bacteria appear on the electrode surface in response to an applied potential

Video 2: [Cable bacteria at poised electrode](https://www.dropbox.com/s/zdrc0c8ynsdb40u/Poised%20electrode.avi?dl=0)

Cable bacteria present abundantly at the poised electrode

Video 3: [Unpopulated unpoised electrode](https://www.dropbox.com/s/ds8f7w2z631jr3m/Unpoised%20electrode.avi?dl=0)

The unpoised electrode did not attract cable bacteria

Video 4: [Cable bacteria pulling at carbon fiber](https://www.dropbox.com/scl/fi/tjlwlwh17vy6bykr5a5x1/VideoS4_CEBBESpaper_AiyerBonn-_PullingCF2_Arrow.mp4?rlkey=7xdxx7xzxq9z6ncnz2z6llf6z&st=2csi3x8z&dl=0)

Cable bacteria pulling the electrode: Cable bacteria attach to the carbon fiber electrode surface and pull it after migrating there. The arrow marks a few moving electrode fibers being pulled by the cable bacteria.

Video 5: [Robustness of cable bacteria attachment to electrode](https://www.dropbox.com/s/ejt0p35tyiynhsp/CB%20Electrode%20Shaking.avi?dl=0)

Robustness of cable bacteria attachment to the electrode

Upon applying a potential, cable bacteria start populating the electrode in a matter of hours (video 1). The poised electrode attracts significantly larger numbers of cable bacteria, which are present on the electrode (video 2 and 3). In the unpoised electrode, this phenomenon is not observed. In video 4 and 5 we demonstrate the direct attachment of cable bacteria on to the electrode.
